# Intracranial compliance is associated with symptoms of orthostatic intolerance in chronic fatigue syndrome

**DOI:** 10.1371/journal.pone.0200068

**Published:** 2018-07-03

**Authors:** Andreas Finkelmeyer, Jiabao He, Laura Maclachlan, Andrew M. Blamire, Julia L. Newton

**Affiliations:** 1 Institute of Neuroscience, Newcastle University, Newcastle upon Tyne, England, United Kingdom; 2 Newcastle Magnetic Resonance Centre, Newcastle University, Newcastle upon Tyne, England, United Kingdom; 3 Aberdeen Biomedical Imaging Centre, University of Aberdeen, Aberdeen, Scotland, United Kingdom; 4 Institute of Cellular Medicine, Newcastle University, Newcastle upon Tyne, England, United Kingdom; Medical Photonics Research Center, Hamamatsu University School of Medicine, JAPAN

## Abstract

Symptoms of orthostatic intolerance (OI) are common in Chronic Fatigue Syndrome (CFS) and similar disorders. These symptoms may relate to individual differences in intracranial compliance and cerebral blood perfusion. The present study used phase-contrast, quantitative flow magnetic resonance imaging (MRI) to determine intracranial compliance based on arterial inflow, venous outflow and cerebrospinal fluid flow along the spinal canal into and out of the cranial cavity. Flow-sensitive Alternating Inversion Recovery (FAIR) Arterial Spin Labelling was used to measure cerebral blood perfusion at rest. Forty patients with CFS and 10 age and gender matched controls were scanned. Severity of symptoms of OI was determined from self-report using the Autonomic Symptom Profile. CFS patients reported significantly higher levels of OI (p < .001). Within the patient group, higher severity of OI symptoms were associated with lower intracranial compliance (r = -.346, p = .033) and higher resting perfusion (r = .337, p = .038). In both groups intracranial compliance was negatively correlated with cerebral perfusion. There were no significant differences between the groups in intracranial compliance or perfusion. In patients with CFS, low intracranial compliance and high resting cerebral perfusion appear to be associated with an increased severity of symptoms of OI. This may signify alterations in the ability of the cerebral vasculature to cope with changes to systemic blood pressure due to orthostatic stress, but this may not be specific to CFS.

## Introduction

Orthostatic intolerance (OI) refers to symptoms that occur when individuals assume an upright posture, often from lying down, which are relieved once the individual returns to a supine posture including light-headedness, dizziness, blurred vision, headaches or sweating. They are accompanied by changes in heart rate or blood pressure. In extreme cases OI can lead to a temporary loss of consciousness (syncope). OI is frequently reported in patients with Chronic Fatigue Syndrome (CFS) or Myalgic Encephalomyelitis (ME)[1]. There is also considerable overlap in symptoms between CFS/ME and specific forms of OI such as postural orthostatic tachycardia syndrome (POTS) [2]. A proportion of CFS/ME patients do have co-morbid POTS [3] [4], although POTS prevalence may not be higher in CFS than in other fatigued patients [5]. However, OI symptoms have substantial intraindividual variability in both CFS/ME [6] and POTS [7], which may complicate precise differential diagnosis.

Due to its high metabolic demand the human brain is particularly vulnerable to blood flow alterations, and various mechanisms act to maintain relatively stable cerebral perfusion despite varying systemic blood pressure [8]. Some of these are believed to be active regulatory mechanisms known as cerebral autoregulation [9]. However, passive mechanical properties of cerebral tissue also play a major part in regulating the blood pressure-flow relationship [10]. Cerebral autoregulation is believed to be most effective in regulating cerebral blood flow at frequencies well below the heart rate, whereas mechanical properties such as vascular resistance and intracranial compliance (ICC) affect cerebral blood flow at higher frequencies [11].

Alterations in peripheral vascular compliance have previously been reported in CFS [12], but it is unknown if this also affects the cerebral vasculature. Given the role of ICC in regulating the cerebral blood pressure-flow relationship and the fact that CFS patients frequently report symptoms of OI, we hypothesize a relationship between OI and ICC in CFS.

CFS is also associated with cerebral hypoperfusion [13–16], though this is not a universal finding [17]. Whether or not this hypoperfusion is related to differences in ICC and may account for symptoms of OI is not yet clear. We hypothesize that reduced cerebral perfusion is associated with increased symptoms of OI in CFS and may be related to ICC.

## Methods

The current study used magnetic resonance imaging (MRI) techniques to measure intracranial compliance and cerebral perfusion in a comparatively large sample of patients with CFS and a sample of age and gender matched control participants. The two groups were compared and the relationship between the MR based measures and OI were examined.

### Participants

A group of 40 patients with CFS (30 female, mean age 45.2 years, range 23–68) participated in this study as part of a larger project investigating the role of autonomic dysfunctions in CFS. Participants with CFS were recruited via the Newcastle and North Tyneside National Health Service (NHS) Clinical CFS Service. Consecutive patients attending the clinic were provided with a Patient Information Sheet and invited to contact the research team if they were willing to be involved. Participants were not selected positively or negatively according to any criteria other than the fact that they were attending the clinical service and had a Fukuda diagnosis of CFS [18], although they were excluded if they screened positive for a major depressive episode or other psychiatric condition other than somatoform and anxiety disorders as assessed using the Structured Clinical Interview for the Diagnostic and Statistical Manual for Mental Disorders [19] (version IV). Other exclusion criteria were use of medication that could affect the autonomic nervous system, pregnancy, substance abuse in the last 6 months, substance dependence in the last year, certain other medical conditions (e.g. diabetes) and contraindications for MRI scanning. Basic characteristics of the patient group are provided in Table 1.

**Table 1:** Participant characteristics.

	CFS group	HV group	group difference
**Age**	45.3 (11.6)	49.4 (15.3)	t = 0.941, p = .35
**Sex (f/m)**	30/10	7/3	χ^2^ = 0.104, p = .75
**BMI**	26.0 (5.1)	26.7 (4.6)	t = 0.428, p = .67
**Mobility level**			
walk unaided	82.5% (33)	100% (10)	
walk with help	17.5% (7)	0% (0)	
not mobile	0% (0)	0% (0)	
**IPAQ**			
Low	57.5% (23)	20% (2)	
Moderate	35.0% (14)	60% (6)	
High	2.5% (1)	10% (1)	
n/a	5% (2)	10% (1)	
MET-score (mean)	725.2 (1006.7)	1403.5 (890.4)	t = 1.854, p = .070
MET-score (median)	198.0	1386.0	U = 83.5, p = .017
**Years of education**	15.5 (3.5)	16.7 (3.6)	t = 0.957, p = .34
**Employment**			
unemployed	62.5% (25)	70% (7)	χ^2^ = 0.195^a^, p = .66
part-time	25% (10)	20% (2)	
full-time	12.5% (5)	10% (1)	
**SF-36 component scores**			
physical health	23.2 (10.1)	53.4 (10.6)	t = 8.340, p < .001
mental health	47.1 (7.0)	55.8 (4.6)	t = 4.769, p < .001
**Duration of symptoms**			
years (mean)	13.0 (9.31)	-	
years (median)	10.5		
**FIS**			
total	90.15 (32.8)	-	
cognitive	22.7 (9.9)		
physical	26.2 (7.6)		
social	41.2 (17.5)		

Unless otherwise noted, values represent mean and standard deviation (in parentheses). BMI = body mass index; IPAQ = International Physical Activity Questionnaire; MET = metabolically equivalent task; SF-36 = Medical Outcomes Study 36-item Short Form Health Survey; FIS = Fatigue Impact Scale.

^a^ compared unemployed vs some employment (part or full-time).

A control group of 10 volunteers (7 female, mean age 49.4 years, range 25–65) were recruited and matched to the patient group in terms of age and sex. Control participants were recruited via notices provided in the hospital and University together with a distribution of posters via the local patient support group. Control participants fulfilled the same inclusion and exclusion criteria as CFS participants other than the fact that they did not have a CFS diagnosis. In addition, controls were required to be sedentary defined as on average not performing more than 30 minutes a week of moderate exercise. This was meant to ensure that any potential differences between the groups would not simply be attributable to differences in levels of everyday activity.

All participants gave written informed consent to the study. The study was conducted in accordance to the Declaration of Helsinki. The study was reviewed and approved by the Newcastle NHS ethics committee (REC 12/NE/0146, CLRN ID 97805).

### Orthostatic intolerance assessment

All participants completed the full Autonomic Symptom Profile (ASP) [20]. The resulting subscales of “Orthostatic Intolerance” and “Syncope” were of most relevance for the present study. As only a small proportion of participants (n = 6) reported any syncope events on the ASP syncope items (1 item, n = 4; 2 items, n = 2), these two scales were combined into a single score (OI+Syn).

### MRI scanning

**Fig 1:**
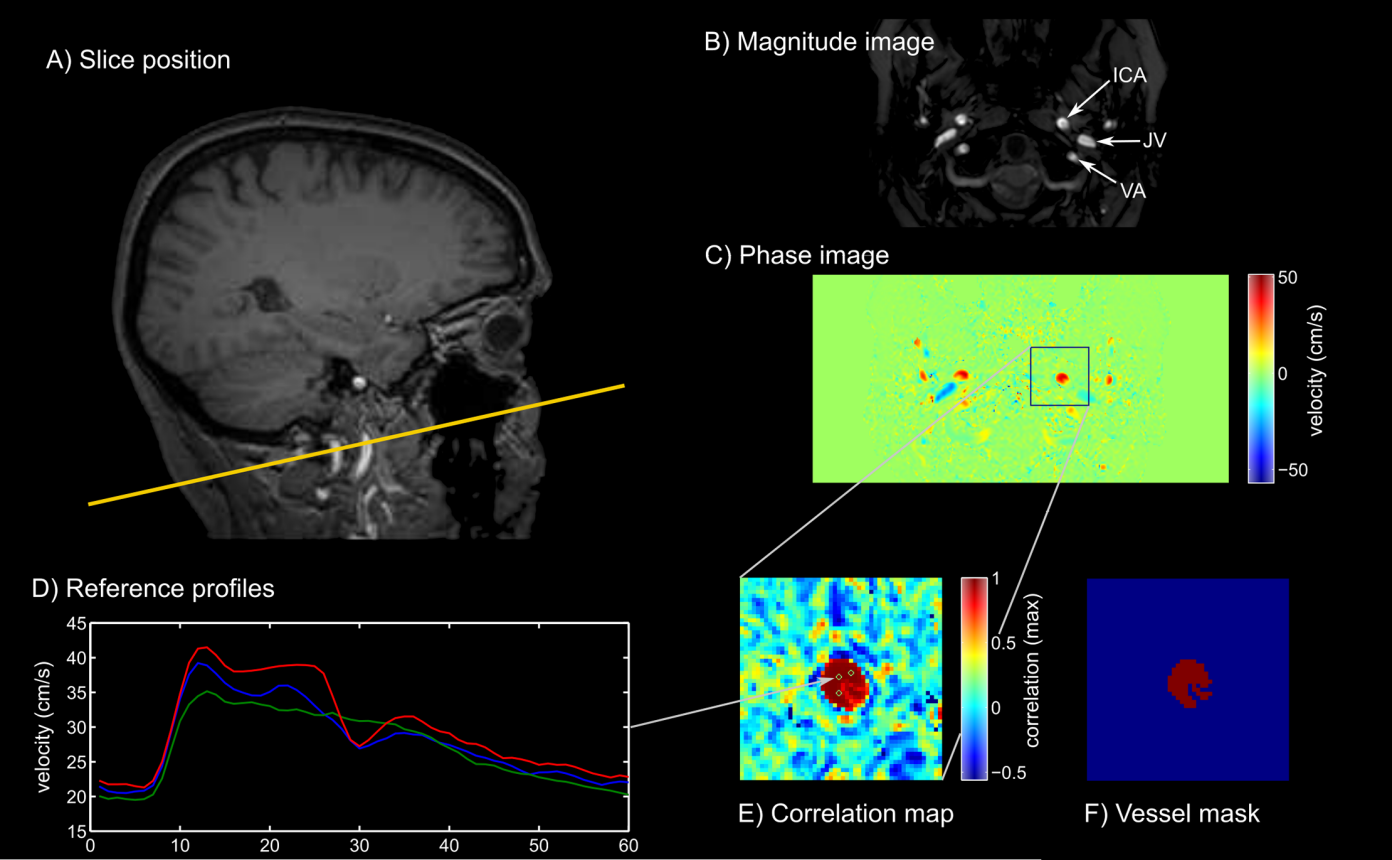
Quantitative flow imaging and data extraction. A) example slice position; B) example magnitude image with position of blood vessels marked on one side (ICA = internal carotid artery, JV = jugular vein, VA = vertebral artery); C) phase image at maximum arterial velocity; D) example velocity profiles of selected reference voxels in right ICA; E) resulting correlation map with positions of reference profiles marked; F) final vessel mask after thresholding of correlation map.

Participants were scanned on a 3T Achieva® (Philips Healthcare, Best, NL) MRI scanner with an 8-channel head coil. In addition to standard, clinical T1- and T2-weighted anatomical brain scans, two sets of scans were performed to assess intracranial compliance and perfusion. For intracranial compliance, a set of three cardiac-gated phase contrast scans were performed to quantify the intracranial volume and pressure change profile across to the cardiac cycle. Scans were collected in a single slice (2D spoiled gradient echo sequence, TR = 9.337ms, TE = 5.64ms, FOV = 200mm x 200mm) positioned at the base of the skull (approximately lower C1, upper C2 vertebrae) to minimize the influence of neck vessel compliance on the measurements [21], and placed perpendicular to the internal carotid and vertebral arteries (see Fig 1A). These scans varied only in the settings of the selected phase encoding velocity through the slice (75 cm/s, 40 cm/s, 10 cm/s), and were chosen to maximize the sensitivity of the measurements to arterial, venous and cerebrospinal fluid (CSF) flow. Sixty phases of the cardiac cycle were acquired with each scan.

Cerebral perfusion was assessed using flow-sensitive alternating inversion recovery (FAIR) arterial spin labelling (ASL) scans. ASL scans utilized variable-rate selective inversion pulses [22] and gradient-echo echo-planar-imaging readout (TE = 12.59, TR = 4000ms, voxel size 4x4x6 mm^3^, 10 axial slices). Sixty tag-control image pairs were acquired with a post inversion delay of 1500ms. Additional inversion recovery scans with inversion times of 900, 1200, 1800, 2100 and 2400ms were acquired for T1 estimation.

### Intracranial compliance analysis

ICC calculations were based on the measured velocities in four arteries (left and right internal carotid, left and right vertebral artery), the left and right internal jugular vein and the CSF flow along the spinal canal as previously described [23]. For each vessel/lumen the set of phase images was selected that offered the highest dynamic range, yet eliminated or minimized the number of voxels exhibiting velocity aliasing in the lumen of interest. In rare cases, voxels with aliased velocity profiles were automatically corrected. The identification of lumen areas used a semi-automatic approach that separated background tissue voxels from vessel voxels based on cross-correlation with manually selected reference flow profiles (Fig 1C–1E) of voxels within each lumen [24]. Cut-offs for correlation coefficients were determined manually for each lumen, aided by visual depiction of the coefficient distribution.

Velocity profiles were extracted for each of the selected voxels. These were then used to calculate arterial inflow, venous outflow, CSF flow and the intracranial volume change profile (ΔV(t) in ml), which measures the compression and expansion of intracranial tissue due to the differential inflow and outflow of fluids to and from the cranial cavity. As only a limited number of blood vessels were assessed, this profile was corrected such that cumulative inflows and outflows over the full cardiac cycle were equal. The overall peak-to-peak volume change was used in the compliance calculation. Flow-velocities of the CSF were used to calculate a pressure gradient profile (ΔP(t)/d) as described elsewhere [25]. The peak-to-peak pressure gradient difference (in mmHg*cm^-1^) of this profile was used to calculate the intracranial compliance index (ICCI), which divided the peak-to-peak volume change by peak-to-peak pressure gradient.

### Cerebral perfusion analysis

ASL images were re-aligned to the first scan and co-registered to the anatomical scan using rigid-body transformations. All subsequent ASL processing was based on extracted signal levels from the grey matter segment, which was derived from a high-resolution anatomical scan using standard segmentation routines implemented in SPM8 (http://www.fil.ion.ucl.ac.uk/spm/). The average tag-control signal level difference of the FAIR scans was used to calculate blood perfusion values according to a standard kinetic model [26]. Additional parameters for these calculations were based on published findings [27] [28] [29].

### Statistical analysis

Statistical analysis was performed in SPSS version 21 (International Business Machines Corp., Armonk, NY, USA). Group comparisons were performed using parametric tests (t-test, ANCOVA), with the exception of ASP scores, which utilized a non-parametric rank test. Within groups, Pearson bivariate and partial correlations were calculated to assess relationships among the measures.

## Results

### Group comparisons

**Fig 2:**
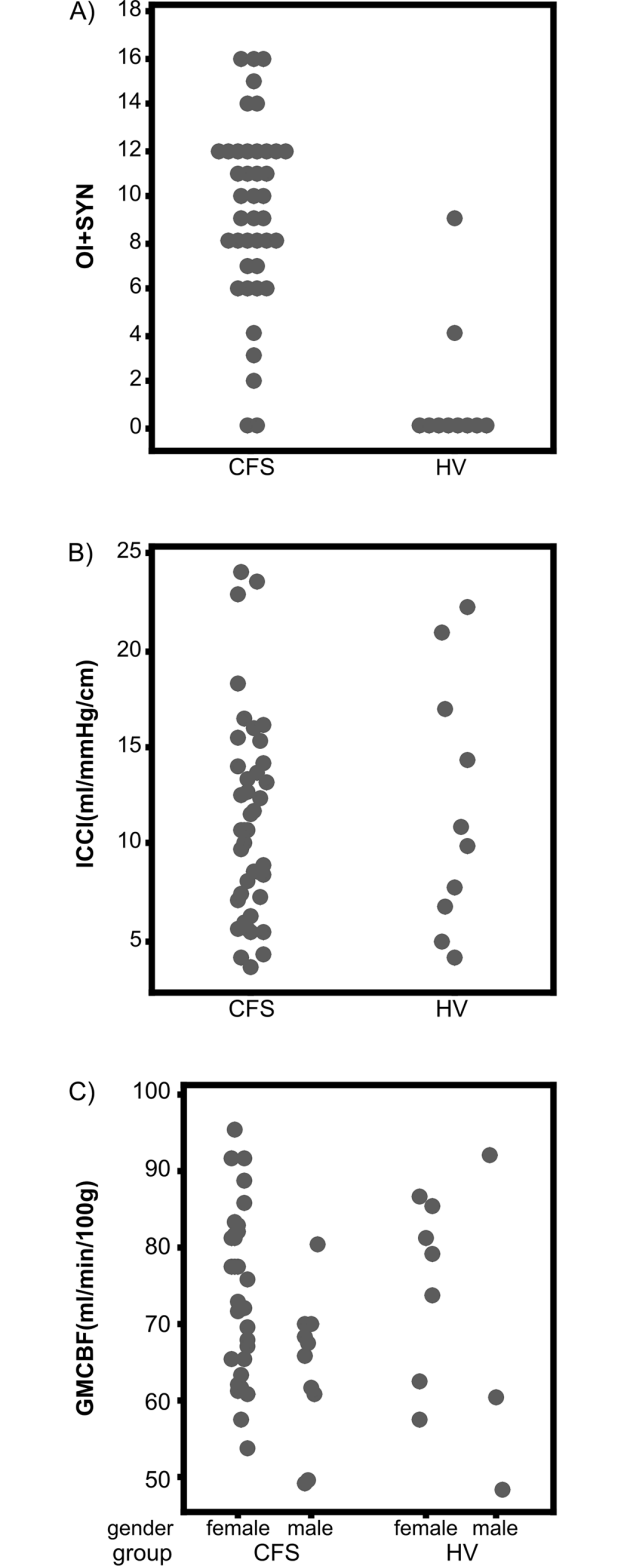
Group comparisons. Group comparisons of orthostatic intolerance (A), intracranial compliance (B) and cerebral perfusion (C).

OI+Syn scores are shown in Fig 2A. As expected, there were clear differences between CFS patients and controls. Only two of 40 patients reported not having any symptoms (i.e. score of 0). The remainder of the patient group reported symptoms ranging from score 2 to score 16. Patients had a mean OI+Syn score of 9.33 (SD = 4.05; median = 10). On the other hand, eight of the ten controls reported no symptoms with the remaining two reporting mild symptoms with scores of 3 and 8 respectively (mean = 1.30, SD = 2.98, median = 0). There was a significant difference in OI+Syn score between the two groups (Mann-Whitney U = 31.0, p < .001).

ICCI are shown in Fig 2B. In the CFS group ICCI ranged from 3.59 to 24.06 ml/mmHg*cm^-1^ (mean = 11.18, SD = 5.22, median = 10.62). In the control group ICCI ranged from 4.08 to 22.31 ml/mmHg*cm^-1^ (mean = 11.83, SD = 6.55, median = 10.28). There was no significant difference in ICCI between the two groups, t(49) = 0.339, p = .74. Grey matter CBF (Fig 2C) ranged from 49.0 to 95.2 ml/100g*min (mean = 71.4, SD = 11.4, median = 69.6) in the CFS group, and from 47.9 to 91.6 ml/100g*min (mean = 72.4, SD = 14.6, median = 76.1) in the control group. Controlling for sex, there was no significant difference between the two groups, F(1,47)<1.

### Relationships between OI, compliance and CBF

Correlations were computed in the CFS group between ICCI, grey matter CBF, the ASP OI+Syn score, age and sex. This showed a negative correlation between OI+Syn and ICCI, r = -.386, p = .013, and a positive correlation between OI+Syn and CBF, r = .417, p = .007. ICCI and CBF also correlated highly, r = -.406, p = .009. Furthermore, age was significantly correlated with OI+Syn, r = -.326, p = .037, and with CBF, r = -.486, p = .001; sex was significantly correlated with CBF, r = -.381, p = .015. Because of the influences of the demographic factors on both orthostatic symptoms and the MRI-based measures, partial correlations were computed controlling for age and sex.

**Fig 3:**
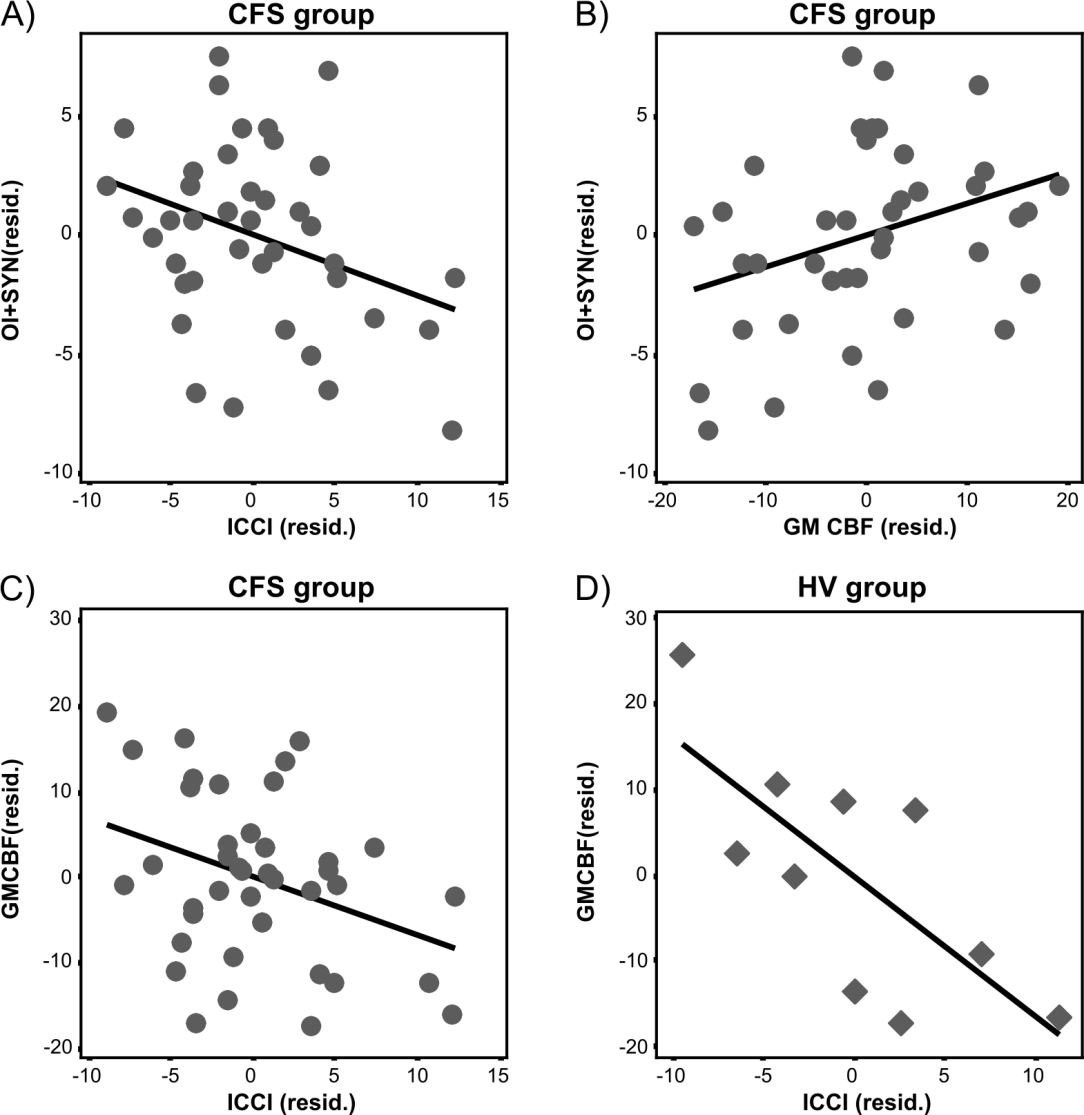
Residual scatter plots. (A) Relationship between OI and ICCI in the patient group. (B) Relationship between OI and CBF in the patient group. (C) Relationship between CBF and ICCI in the patient group, and (D) the control group. All scores were adjusted for effects of age and sex.

Within the patient group, the partial correlations between OI+Syn and ICCI, r = -.346, p = .033, between OI+Syn and CBF, r = .337, p = .038, and between CBF and ICCI, r = -.337, p = .036, were all significant. In controls, there was a negative partial correlation between ICCI and CBF, r = -.737, p = .037. Fig 3 shows scatter plots of these relationships (using residual plots after accounting for influences of age and sex).

As ICC is measured as a ratio of changes in intracranial volume and pressure, we explored if these two components were individually associated with OI in the patient group. Covarying for age and sex, there was a trend for a positive relationship between the peak-to-peak pressure gradient and orthostatic symptoms, r = .302, p = .065, but not between peak-to-peak volume change and orthostatic symptoms, r = -.146, p = .38. The peak-to-peak pressure gradient itself was negatively correlated with diastolic blood pressure at supine rest, r = -.352, p = .030. However, blood pressure itself was unrelated to orthostatic symptoms (p = .57 for systolic and p = .74 for diastolic blood pressure) or ICCI (systolic p = .46, diastolic p = .74).

## Discussion

The current study indicates a potential role of ICC in the severity of symptoms of OI in patients with CFS. As expected, almost all CFS patients in our sample reported various levels of OI via self-report that were significantly higher than those reported by the healthy control group. In the patient group there was a negative relationship between OI symptom severity and ICC, as those patients with higher compliance tended to report lower OI symptoms (and vice versa). Furthermore, there was a positive correlation between orthostatic symptoms and cerebral blood perfusion in grey matter. This suggests that CFS patients who report more severe orthostatic symptoms have higher levels of resting cerebral perfusion than those patients who report fewer or only milder orthostatic symptoms.

While, as expected, orthostatic symptom severity was significantly higher in the patient group than in healthy controls, ICC did not differ between the two groups. A previous study that used very similar MR methods was also unable to find differences between CFS patients and controls in pulsatile CSF flow parameters [30]. Similarly, there was no significant difference between the groups in grey matter cerebral perfusion. This contradicts several earlier findings that have reported reduced perfusion in CFS patients [16, 31, 32]. However, reports of cerebral hypoperfusion are not universal as other studies have also reported regional cerebral hyperperfusion in CFS [33]. It is important to note that the present study only investigated average grey matter perfusion across the whole brain and it cannot be excluded that regional hypo- or hyperperfusion were present. Furthermore, not all of the above studies have excluded CFS patients with comorbid depression, whereas the current study did so explicitly. As depression has been associated with cerebral hypoperfusion[34], some of the reported findings may in part be due to depression and not chronic fatigue.

While the relatively small size of our control group may have prevented us from identifying group differences in compliance or perfusion, even the mean values were numerically very close to each other so that a simple lack of statistical power is unlikely be the sole reason for not finding group differences. It should however be noted that our control group was deliberately recruited to be mostly sedentary and therefore may not have been at optimum levels of general health itself. Furthermore, the relationship between ICC and cerebral perfusion appeared to be very similar in both groups. This suggests that ICC and cerebral perfusion were not altered in our CFS patients relative to the control group. Instead it appears that low intracranial compliance, which was strongly negatively coupled to higher resting perfusion, is associated with experiencing higher severity of symptoms of OI in the CFS group if the cardiovascular system is not capable of providing adequate blood supply to the brain due to an orthostatic stressor.

The negative relationship between ICC and cerebral perfusion may seem puzzling at first. However, given that the ICCI is inversely proportional to the peak-to-peak pressure gradient, lower compliance may be an expression of larger pressure gradients, which may increase blood perfusion. Indeed, in patients there is a significant positive correlation between CBF and the peak-to-peak pressure gradient, but not with peak-to-peak intracranial volume change. In contrast, CBF in controls showed a significantly negative correlation with intracranial volume change (r = -.826, p = .012), but no significant correlation with peak-to-peak pressure gradient (r = .468, p = .24). In other words, the negative relationship between ICC and cerebral perfusion appears to be primarily determined by pressure differences in patients, whereas it is primarily determined by volume changes in controls.

It should also be noted that CBF and compliance measurements were made at rest while participants were in a supine position on the MR scanner bed. A study in healthy volunteers using a low-field, vertical gap MRI scanner and comparing a supine to an upright posture showed that these parameters are indeed posture dependent [35]. It is conceivable that the supine posture of the current study had increased cerebral perfusion in low compliance, high OI symptom patients, and that their cerebral perfusion measurements would have been similar to high-compliance/low OI symptom patients had the perfusion measurement been taken in an upright position. While it is not possible to dynamically change a subject’s position from supine to upright in current high-field MR scanners, other techniques such as lower-body negative pressure or thigh-cuff deflation could be utilized in future studies to simulate different orthostatic conditions and their effect on cerebral perfusion.

However, studies using transcranial Doppler ultrasonography showed that patients with POTS and healthy controls have near identical blood flow velocities in the middle cerebral artery, which decreased equally with increasing levels of tilt [36, 37]. Instead, POTS patients showed an increase in low-frequency oscillatory blood flow with increasing levels of tilt, whereas controls did not. The authors argued that these increased low-frequency oscillations are an expression of impaired cerebral autoregulation and that they might interfere with normal neurovascular coupling [37]. Low ICC in our high-OI patients may have put a limit on their ability for dynamic cerebral autoregulation, thereby resulting in similar low-frequency blood flow oscillations. Furthermore, as (intracranial or vascular) compliance is directly responsible for dampening BP oscillations at high frequencies (at and above heart rate), low ICC could increase the amount of pressure oscillation that is transmitted to perivascular cerebral tissue at these higher frequencies and might cause similar interference with normal neurovascular coupling as those hypothesized for low-frequency oscillations.

In summary, we present evidence for a relationship between the degree of self-reported severity of orthostatic intolerance in patients with chronic fatigue syndrome and MRI based measures of intracranial compliance and cerebral blood perfusion. Patients with more severe OI symptoms exhibited lower levels of ICC and higher levels of cerebral perfusion. While neither of those measures was significantly different from the control group and an inverse relationship between compliance and perfusion was observed in both CFS patients and in controls, reduced ICC may constitute a risk factor for experiencing symptoms of orthostatic intolerance as it might reduce an individual’s ability for effective cerebral autoregulation. Whether the supine posture in the MR scanner had elevated cerebral perfusion in individuals with low compliance remains unclear and should be the subject of further investigation. Future studies should attempt to replicate these findings perhaps using more (clinically) accessible methods such as transcranial Doppler ultrasonography. If a link between the severity of orthostatic symptoms and ICC can be replicated, this would suggest that patients who experience severe symptoms of OI should be investigated for low levels of ICC. It could then also be investigated whether treatments that raise ICC have ameliorating effects on orthostatic symptoms.

## Supporting information

S1 File Manuscript data. (XLSX)
